# Deaths and Severe Adverse Events Associated with Anesthesia-Assisted Rapid Opioid Detoxification — New York City, 2012

**Published:** 2013-09-27

**Authors:** David Berlin, Brenna M. Farmer, Rama B. Rao, Joseph Rella, Hillary Kunins, Deborah Dowell, Nathan Graber, Robert S. Hoffman, Adam Karpati, Don Weiss, Christopher Jones, Amita Toprani, Alison Ridpath

**Affiliations:** New York Presbyterian Hospital Weill Cornell Medical Center; New York City Dept of Health and Mental Hygiene; Div of Unintentional Injury Prevention, National Center for Injury Prevention and Control; EIS officers, CDC

During August–September 2012, the New York City Department of Health and Mental Hygiene (DOHMH) was notified by the New York City Poison Control Center regarding three patients who experienced serious adverse events after anesthesia-assisted rapid opiate detoxification (AAROD) at a local outpatient clinic. All three patients required hospitalization, and one subsequently died. DOHMH issued an order requiring that the clinic cease performing AAROD pending an investigation and searched for additional cases of AAROD-related serious adverse events at the clinic and elsewhere in New York City for the period September 2011 to September 2012. That search found no serious adverse events at clinics other than the one implicated. Of the 75 patients who underwent AAROD at the implicated clinic during January–September 2012, two died, and five others experienced serious adverse events requiring hospitalization. As a result of the findings, the New York State Department of Health, the New York Office of Alcoholism and Substance Abuse Services, and DOHMH jointly issued a Health Alert informing New York health-care providers of AAROD-associated serious adverse events and recommending that they avoid use of AAROD in favor of evidence-based options for opioid dependence treatment.

## Health Department Investigation

AAROD procedures performed in the New York City clinic included 1) administration of medications (e.g., clonidine, antiemetics, and antidiarrheal agents) that blunt withdrawal symptoms, 2) intubation and induction of general anesthesia, 3) precipitation of opioid withdrawal by intravenous infusion of high doses of the opioid antagonist naloxone or intramuscular injection of naltrexone, 4) maintenance of anesthesia until withdrawal symptoms were presumed to have subsided, and 5) extubation and monitoring during an overnight recovery. Median duration of anesthesia was 8.3 hours (range: 3.1–15.0 hours); median duration of opioid antagonist infusion was 3.9 hours (range: 2.1–14.0 hours). Median naloxone dose was 80 mg (range: 2–315 mg); median naltrexone dose was 133 mg (range: 25–300 mg). For patients with serious adverse events, the median naloxone dose was 80 mg (range: 4–88 mg) and median naltrexone dose was 150 mg (range: 0–150 mg). All patients were monitored overnight after the procedure.

A serious AAROD-associated adverse event was defined as hospitalization for any cause or death <72 hours after undergoing AAROD in New York City during September 1, 2011–September 5, 2012. DOHMH staff conducted two visits to the clinic. All four clinic staff members were interviewed, and medical records for all patients who underwent AAROD while the clinic was operational were reviewed. Records of emergency medical services calls to the clinic were obtained from the New York City Fire Department. Hospital records for all patients who were found to have made emergency department visits or been admitted to a hospital were reviewed. The practice’s patient list was matched to mortality records by patient name and date of birth in New York City and the patients’ usual states of residence. New York City’s Poison Control Center toxicology database was searched for serious adverse events from other New York City health-care facilities.

No emergency medical services calls to the practice were reported other than those initially reported by the Poison Control Center. The mortality records and toxicology database searches yielded no additional AAROD-related serious adverse events from the implicated clinic or elsewhere. From the clinic’s opening in January 26, 2012, until September 4, 2012, a total of 75 patients underwent AAROD; 62 (83%) were men (median age: 37 years; range: 20–63 years). Patient comorbidities included psychiatric disorders (55%), chronic medical conditions (23%), and polysubstance use (35%). In addition to the three adverse events reported, four additional adverse events, including one additional death, were identified during medical record review. All seven patients were men (median age: 31 years; range: 24–52 years), Four were prescription opioid users; two used both prescription opioids and heroin, and one used heroin alone. Four patients had psychiatric comorbidities, and two were polysubstance users. None of the patients had a documented chronic medical condition.

## Case Reports

### Case 1

On April 14, 2012, a man aged 52 years underwent AAROD. The next evening he experienced vomiting and weakness and was admitted to the hospital with a temperature of 104°F (40°C) and a white blood cell count of 26 x 10^3^ cells/*μ*L (normal range: 3.9–10.7 x 10^3^ cells/μL). He was treated empirically for sepsis and discharged on April 18.

### Case 2

On April 16, 2012, a man aged 23 years with a history of depression and panic attacks underwent AAROD; during the recovery period he experienced two panic attacks and was administered benzodiazepines. The next day he was admitted for inpatient stabilization after displaying violent behavior and expressing suicidal thoughts. He was discharged on April 25 with stable mental status.

### Case 3

On June 3, 2012, a man aged 30 years underwent AAROD. On extubation, he was unable to speak or follow commands. Eight hours after extubation, he was transported from the clinic to an emergency department, where he was found to have pulmonary edema. He was admitted to the intensive care unit and intubated after an episode of emesis with aspiration. He was treated for aspiration pneumonia, extubated on June 6, and discharged on June 11 with normal mental status.

### Case 4

On July 20, 2012, a man aged 46 years with a history of heroin, cocaine, and alcohol abuse underwent AAROD. Urine toxicology on that day revealed trace amounts of cocaine. He was discharged on July 21. He was found dead by his wife at approximately 10 a.m. on July 22 after leaving the bedroom at approximately 4 a.m. and telling his wife that he was going to take something for abdominal pain. Autopsy results indicated pulmonary edema and cardiomegaly.

### Case 5

On August 19, 2012, a man aged 31 years underwent AAROD. The next day he experienced diarrhea, weakness, and blurry vision. On hospital admission he had hypokalemia (2.9 mEq/L [normal range: 3.5–5.0 mEq/L]) and elevated creatine kinase concentrations (1,346 U/L [normal range: 30–170 U/L]). He was treated for rhabdomyolysis and electrolyte abnormalities and discharged on August 22.

### Case 6

On August 23, 2012, a man aged 51 years underwent AAROD. Approximately 10 hours after extubation, while being monitored at the clinic, he experienced cardiac arrest with ventricular fibrillation. He was resuscitated and transferred to a hospital. At the hospital, his serum potassium was 2.6 mEq/L (normal range: 3.5–5.0 mEq/L). Computed tomography revealed cerebral edema. He experienced brainstem herniation and was pronounced dead on September 1. Autopsy revealed anoxic encephalopathy and marked coronary atherosclerosis; the cause of death was “hypokalemia and cardiac arrhythmia following anesthesia-assisted rapid opiate detoxification.”

### Case 7

On September 4, 2012, a man aged 26 years underwent AAROD. Approximately 30 minutes after naloxone infusion was initiated, he experienced cardiac arrest. He was resuscitated and transported to a hospital. His hospital course was complicated by necrotizing fasciitis of the right arm, for which he underwent surgical debridement before discharge on September 25.

What is already known on this topic?

Anesthesia-assisted rapid opiate detoxification (AAROD) does not reduce subjective opioid withdrawal symptom scores more than traditional opioid detoxification modalities, but has been associated with a high risk for severe adverse events, including death.

What is added by this report?

Of 75 patients who underwent AAROD at a New York City clinic during January–September 2012, two died and five others experienced serious adverse events requiring hospitalization.

What are the implications for public health practice?

To reduce the morbidity and mortality associated with opioid dependence, evidence-based approaches (e.g., medication-assisted treatment) should be used for its management.

### Editorial Note

Opioid abuse and dependence is a serious public health problem in the United States. During 1999–2008, emergency department visits, overdose deaths, and substance abuse treatment admissions related to prescription opioids increased substantially (1). Opioid dependence is a chronic and relapsing illness. Evidence-based treatment options include medication-assisted treatment (MAT) with long-acting opioid agonists (e.g., methadone or buprenorphine), maintenance treatment with opioid antagonists (e.g., naltrexone), or counseling and behavioral interventions (2,3–5). Treatment goals include long-term abstinence or reduction in illicit and nonmedical drug use. MAT is considered first-line treatment among the evidence-based options listed previously and, compared with other treatments, is associated with lower mortality, improved treatment retention, and decreased incidence of comorbid illnesses, including human immunodeficiency virus infection (2). However, MAT treatment capacity is insufficient to meet demand in the United States, and patients frequently are placed on waiting lists (6).

Opioid detoxification refers to the discontinuation of opioid use under medical supervision and includes prescribing or administering medications to decrease withdrawal symptoms. Standard detoxification methods include administering gradually reduced doses of long-acting opioid agonists during a 3–21 day period or discontinuing opioids and administering nonopioid medications to block withdrawal symptoms. These methods ameliorate withdrawal symptoms and carry <1% risk for serious adverse events (3,4). The effect of detoxification on long-term abstinence is negligible without the addition of longer term evidence-based substance abuse treatment (5). Medically supervised opioid detoxification, however, when closely associated with substance abuse treatment programs, can provide an entry point to care.

AAROD was developed during the 1980s with the goal of reducing the discomfort of withdrawal and thereby encouraging patients to enter substance abuse treatment. However, AAROD and standard opioid detoxification do not differ in subjective withdrawal symptom scores or in achievement of short-term abstinence (7). Few long-term studies of AAROD exist, but published data indicate that AAROD does not improve 12-month abstinence rates, compared with standard detoxification (7). Furthermore, AAROD is associated with a substantial rate of serious adverse events in the research setting, 8.6% in one study (8).

Government agencies and professional societies,* including the American Society of Addiction Medicine, have recommended against using AAROD in clinical settings (9). There is insufficient knowledge regarding how widely AAROD is used in the United States and the frequency of AAROD-associated adverse events in community practice settings. At least seven deaths occurred following AAROD among 2,350 procedures performed in one practice during 1995–1999.†

The New York City clinic investigation revealed that AAROD was performed on 75 patients during January–September 2012 and was associated with two deaths and five additional adverse events requiring hospitalization, a serious adverse event rate of 9.3%. No standard protocol exists for AAROD; however, the clinic’s practice was consistent with AAROD use described elsewhere (7). All events occurred after and in close temporal proximity to AAROD. Although a common mechanism linking these events to AAROD is not evident, the events are consistent with previously proposed mechanisms of AAROD-associated adverse events, including electrolyte disturbance, catecholamine release, altered cardiopulmonary functioning, acute lung injury, and other physiologic effects associated with administration of high doses of opioid antagonists under general anesthesia (10). Given the ongoing epidemic of prescription opioid dependence, further increases in the demand for substance use disorder services are to be expected. AAROD has substantial risks, including a risk for death, and little to no evidence to support its use. Safe, evidence-based treatments of opioid dependence (e.g., MAT) exist and are preferred (2).

## Footnotes

* Additional information available at http://www.nice.org.uk/cg052.

† Additional information available at http://njlaw.rutgers.edu/collections/oal/final/bds10905-99_2.pdf.
